# Designing Nanoparticle-based Drug Delivery Systems for Precision Medicine

**DOI:** 10.7150/ijms.60874

**Published:** 2021-06-05

**Authors:** Jianhua Yang, Chengyou Jia, Jianshe Yang

**Affiliations:** 1 Department of Orthopaedics, Longgang District People's Hospital, Shenzhen 518172, China.; 2 Shanghai Research Center for Thyroid Diseases, Shanghai Tenth People's Hospital, Tongji University School of Medicine, Shanghai 200072, China.; 3 Health and Life Science College, the Chinese University of Hong Kong, Shenzhen 518172, China.

**Keywords:** nanoparticles, drug delivery system, precision medicine, drug design

## Abstract

Traditional drugs are facing bottlenecks of lower solubility, absorption, and especially the inefficient organs or cells targeting during the precision medicine era. It is urgently needed to discover and establish new methods or strategies to modify old drugs or create new ones against the above defects. With the support of nanotechnology, the solubility, absorption and targeting of traditional drugs were greatly improved by modifying and fabricating with various types of nanoparticles to some extent, though many shortages remain. In this mini-review we will focus on advances in several most commonly used nanoparticles, from their nature and design, to drug delivery system and clinical application, that they overcome heterogeneous barriers in precision medicine, thereby ultimately improve patient outcome overall.

## Introduction

From a scientific perspective we know the engineering nanomaterials have significant priority to promoting disease diagnosis and treatment. Nanotechnology can facilitate the drug delivery through nanomaterials targeted modification, transport of desired molecules to specific organelles. The nanotechnology program has thrived worldwide since 20 years ago, such as the US National Science and Technology Council (NSTC) launched the National Nanotechnology Initiative (NNI) in 2000, and presented clear plans and major challenges in this field. Nanoparticles (NPs) occupy a large part of all the plans with the source variation and preparation diversity. What-forever, the improvement in stability and solubility of the NPs depends on many factors including the NP composition, formulation, chemical structures of the small drug molecules and its carriers, temperature, pH, etc., thus improving the safety and effectiveness 1, 2. Owning these priorities, NPs have been widely used and produced promising results both in pre-clinical and clinical stages 2-5, 7, 8. As nanoparticle-based precision therapies are applied in a broad area, such as cancer therapy, immunotherapy, and recently in virus infected disease, a full span and timely understanding of NPs progress is particularly important. Here we will report and review several most commonly used NPs in the terms of its preparation and precision medicine application, and expect to afford the guidance to optimize the strategies from its design to clinical application.

## Lipid-based NPs

**Figure 1:**
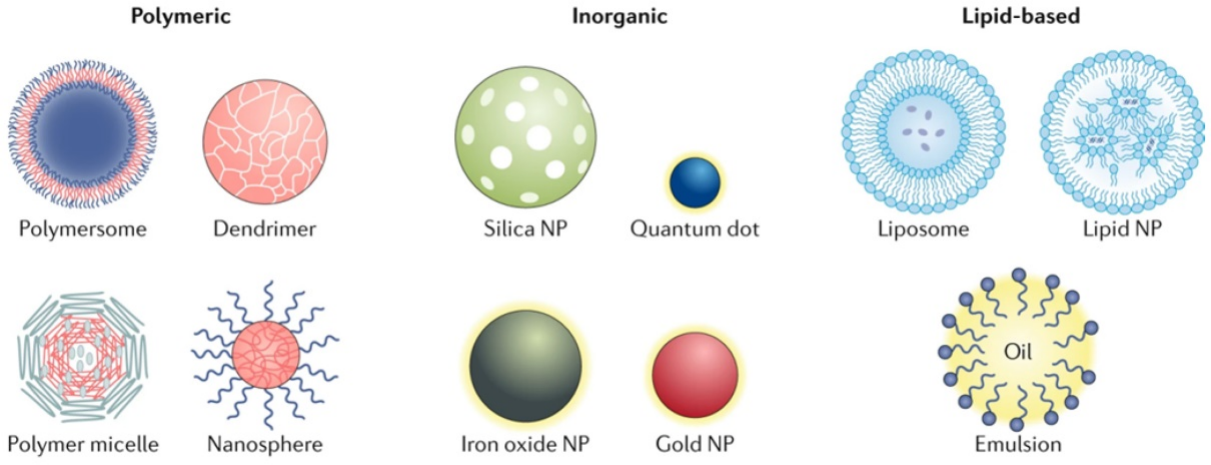
Different types of the nanoparticles. NP-based drug delivery systems can be classified into different types including polymeric, inorganic and lipid-based vehicles 4.

Lipid-based NPs can be designed into various subpopulation structures, among them the spherical structures is most typical. This type of lipid-based NPs is constituted of at least one lipid bilayer surrounding an interior water chamber (Figure 1) 4. Also the lipid-based nano-micelles is of well promise. Lipid-based NPs is regarded as an important drug delivery system relying on its sorts of advantages: simple formulation, self-assembly, well biocompatibility and high bioavailability, large payloads, and improvable physicochemical properties utilized to regulate their biological applications. 5 Thus the lipid-based NPs is the most frequent selection among FDA approved nanomedicine and other countries worldwide 6, 7.

Liposome is one of the most typical lipid NPs subsets, which are the monolayer and multilayer vesicles composed of phospholipids. This special structure allows liposomes convenient to carry and deliver drugs with difference characters from hydrophilic, hydrophobic to lipophilic, and they can even adsorb hydrophilic and lipophilic compounds simultaneously, thus making it more versatile 8. The stability of liposomes whether *in vitro* or *in vivo* is influenced by NPs size, surface charge, lipid component, and surface modification (ligand or polymer), respectively 9. Liposomes are usually modified on its surface to expand their fluidity and enhance drug delivery, and targeting as well. Unmodified liposomes are cleared by RES, while the surface modifications help to avoid the RES and thus prolongs the blood circulation time 10.

Another notable lipid-based NPs subgroup is the lipid nanoparticles widely used for nucleic acid delivery (lipid nanoparticles, LNPs). The micelle structures mode makes LNPs different from traditional liposomes. This formation is within the particle core but not outer, and its morphology can be controlled and modulated according to the formulation and synthesis parameters, and the special formulation and morphology of these LNPs make its with serum stability and could not create compounds with human serum albumin 11. There are commonly four main LNPs components: cationic or ionizable lipids, phospholipids, cholesterol, and polyethylene glycol lipids 12-14. For LNPs, the effectiveness of nucleic acid delivery and its easy synthesis procedure, small volume (0.5-1 micron), and stability in circulation system make it very important to personalized gene therapy applications. The ionizable LNPs are ideal carriers in nucleic acid therapies because they are near neutral charges at physiological conditions, whereas charges in acidic inclusions can facilitate the escape to intracellular release 15.

Nevertheless, despite these advantages, the LNPs system is still limited due to the low drug load and biodistribution, leading to high uptake in the liver and spleen, and result in the acute cumulative drug injury. Improving the drug load capability and targeted uptake are deserved to be resolved immediately.

## Polymer NPs

Polymer NPs can be synthesized using natural or synthetic materials and form various possible structures and features (Figure 1). A variety of techniques were used for the synthesis of polymer NPs, such as emulsification (solvent replacement or diffusion), nano-precipitation, ionic gelation, and microfluidics, all of which yielded different end products 16, 17. Polymer NPs also have variable drug delivery capabilities which depends where and how does the drugs combine with NPs, for instance, drugs can be encapsulated within the NPs nucleus, embedded into the polymer matrix, bound to the polymer through chemical reactions or bonded to the NPs surface. Polymer NPs is an ideal material for drug co-delivery. The loaded drugs can be hydrophobic and hydrophilic compounds or other small molecules and biological macromolecules, such as RNAs/DNAs, proteins and anticancer drug paclitaxel 17, 18. By regulating the chemical and physical properties of NPs and drug can realize the accurate control of the loading effect and release kinetics 18.

There are two usual NPs polymers forms: nanocapsules (cavities surrounded by polymer membranes or enclosures) and nanospheres (nanospheres, solid matrix systems). NPs can be further divided into polymeric vesicles, micelles and dendrimers.

Polymeric vesicles are similar with liposomes, but more stable and efficient for drug retention due to its relative closed structure, thus they are more effective to deliver therapeutic drugs to the cytoplasm 19. Polymeric vesicles commonly used for these uses include polyethylene glycol (PEG) and polydimethylsiloxane (PDMS).

Micelles are the nanospheres with hydrophilic inner core surrounded by hydrophobic coating through polymer NPs self-assembling, which can protect the transport of waterborne drugs and improve the cycling time. This type nanosphere can load various drugs, especially some biommolecules used in clinical trials of cancer therapy 20.

Dendrimers is a highly dendritic polymer with complex three-dimensional structures with highly controlled physical and chemical parameters. The active functional groups outside the dendrimers are prone to be fabricated with biomolecules with targeted tendency, while therapeutic drugs can be loaded inside. The loading capacity of dendrimers is promising, whereas research interest is focusing on delivering the RNA, DNA and proteins. In these applications, some charged polymers, such as polyethylenimine (PEI) and polyaminoines (PAM), are commonly used. Some clinical trials currently ondergoing just select several tree-like polymer products (described in reference 21 and 22) as test materials which owning the anti-inflammatory, transfection functions, also they are used as topical gels and contrast agents 21, 22.

Overall, polymer NPs is an ideal drug delivery material due to its biodegradability, water solubility, biocompatibility, biomimeticity and storage stability. Their all-round surfaces targeted modifications enable them to transport diverse materials to the target tissue and play an important role for diagnosis and treatment in precision medicine. However, due to the high degree of polymerization of polymer NPs, some disadvantages will lead to the risk of particle aggregation and toxicity, fewer polymer nanopharmaceuticals have the clinical license by FDA, only a large number of clinical trials are permitted 23.

## Inorganic NPs

To date, some inorganic materials are prepared into nanostructured materials by monomer or by chemical doping with other matrix for application on drug delivery and imaging. The inorganic nanomaterials are designed into various sizes, structures and geometries. Among them, gold nanoparticles (AuNPs) are of the most frequently been studied with its different form like nanospheres and nanorods, nanoshells 24. Furthermore, the unique electrical, magnetic and optical nature of inorganic NPs (such as Au NPs, quantum dots and iron oxide), decided by the substrate properties, endowing them with many potential effects, like photothermal transforming effect to be a promising application for thermal-therapy 25.

Iron oxide is another common inorganic NPs and accounts for the vast majority of clinical studies in the world, including prognostic imaging and nano-based raditherapy for metabolic diseases and cancers. The magnetic iron oxide NPs consists of Fe_3_O_4_ or Fe_2_O_3_ with superparamagnetic properties in some sizes and has been successfully applied in contrast agents, drug delivery carriers, and therapy 26, 27. Calcium phosphate and mesoporous silica are the other important inorganic NPs used to transport DNA/RNA or drugs 28.

These types of inorganic NPs have unique magnetic, radioactive and plasmonic properties, which make them qualified for clinical diagnosis, imaging, and photothermal therapy. Most of them have good biocompatibility and stability. Cell and animal experiments have demonstrated these metal cores of NPs of very low cytotoxicity, and kept stable status during the course of intake and targeting to the organs with few interactions with surroundings. While, essential effort should be made to promote the solubility and reduce toxicity of these materials, especially when the heavy metals were enrolled into preparation system.

## Carbon NPs

**Figure 2:**
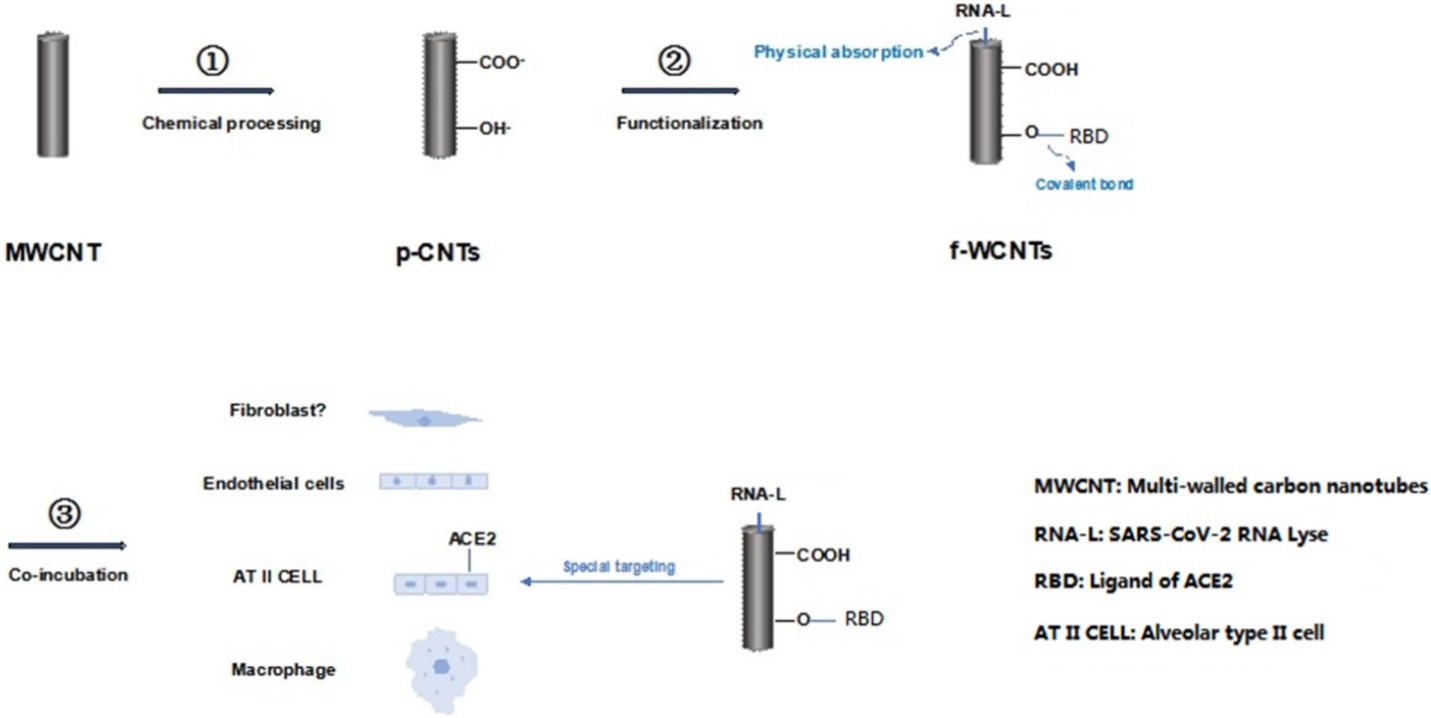
Flow chart of preparation of functional multi-walled carbon nanotubes. This flow chart described briefly the MWCNT was fabricated with strong acid and base conditional mixture in order to achieve the p-WCNT (chemical process); then modified with RNA layse and receptor binding domain (RBD) by covalent conjugation and physical absorption to get f-WCNT (functionalization); thereafter, f-WCNT was used in the multi-cell culture system interacting with SARS-CoV-2 to identify the special affinity of f-WCNT to ACE2 labeled alveolar type II cells and the inhibition capacity to SARS-CoV-2 31.

Carbon nano-materials include a various types of which are containing sole carbon skeleton, such as graphene, fullerene and the most used carbon nanotubes. Due to the rigid structure and low water-solubility, these materials have ever been neglected for biological application. While the unique chemical and physical characteristics of these materials presented after a series of special modification. In our previous studies, we explored the potential value of carbon nanotubes for its radiosensitizer role 29, and a surprised effect for inhibiting the prostate cancer cells without depending on the hormone-deprived therapy 30, even in the novo antiviral nano-medicine intermediate for SARS-CoV-2 prevention (Figure 2) 31.

## NPs in precision medicine

The term “precision medicine” has evolved from “personalized medicine”, which is based on the specific disease with individual unique genetic and molecular profile referred to a fully personalized treatment plan 32. Years after the Human Genome Project finished, we consider prudently that “personalized” is too ambitious and restrictive, and find a substantial amount of relevant information, notable clinical parameters and environmental factors are difficult to be counted by this word anymore. However, the term “precision medicine” greatly expands the practical meaning which including the development of specific drugs and medical practice to a well-defined groups of individuals, both on their susceptibility to a particular disease and probability of responding to a given treatment plan. Consequentely any intervention including primary preventive and comprehensive treatments could be used to the specific subpopulations and they will definitely benefit from these targeted intervention, resulting in maintaining the resources management and systemic equities at high level as possible 33, 34.

Precision medicine can improve the clinical treatment method to minimize the limitations of the conventional therapies. For example, the biomarkers identification and concomitant diagnostics have been a standard in oncology diagnostics and treatments. Without the aidance of these techniques, anti-cancer nanopharmaceuticals will be difficult to work 35.

NPs designed for specific patient groups can break the limitations of current drug delivery, absorption and metabolism, and ultimately qualify more patients get benefit from precision medicine 36.

Applications of nanomaterials in precision medicine have emerged since the launch of the Precision Medicine Initiative (PMI) in 2015 37. An early clinical detection for personalized biomolecular adsorbed on graphene oxide nanosheets was exerted with pancreatic cancer patient blood in 2019 38. Graphene oxide can combine the unique characteristics of a small amount of albumin to enable strong adsorption of low-level proteins present in plasma. Other magnetic NPs or AuNPs showed the priority in biomarker detection analysis with much less time and consumes 39.

Beside the diagnostic usage, some of NPs can reshape the tumor microenvironment, thereby enhancing the sensitivity of tumors to specific treatments 40. Endothelial cells of tumors can be manipulated by the microRNA delivered by NPs, therefore alters the vascular system microenvironment and makes the tumor much more sensitive to conventional cancer therapies. Similar bioinspired lipoproteins have been used to reshape tumors and increase NPs accessibility to cancer cells by 27 times 41. The use of photothermal NPs can improve the wettability and anti-solid tumor activity of CAR-T cells 42 or lead the direct physical destroy to tumors or viruses 30. NPs can also regulate immune function and make cancer cells sensitive to treatment, homogenize environments, and make more patients respond to accurate treatment 43.

Based on the above description, in Table 1, some examples of nanoparticle-based drug delivery systems that are on market or in clinical trials have been presented, like COVID-19 vaccine, DaunoXome, Doxil, Feraheme, etc., along with their delivery systems (liposomes, polymers, metals, or others), active ingredients, target diseases' names, and so on.

## Conclusions

To sum up, the combination of NPs and precision medicine promotes the development of these two fields. Accurate medical classification of patients can accelerate the clinical transformation of many NPs developed for specific patients. Conversely, NPs by improving the delivery and efficacy of precision drugs, more patients are included in the classified population, thus increasing the success rate of precision medicine and benefiting patients. Application of NPs for precision medicine is of precisely design and control. The carefully designed method was able to tune the pharmacokinetics of the therapeutic drug to meet the requirements of solubility, administration or biodistribution and has been successful in the study. Yet the three challenges still need to be solved: (1) detailed and optimized synthesis process for controllable and renewable nanoparticle; (2) found the standard system for clinical evaluation of NPs; (3) implementation of good manufacturing practices (GMP), develop a practical clinical transition plan to achieve mass production.

## Figures and Tables

**Table 1:** Some examples of NPs applied in clinic and clinical trials

Drugs	Company	Materials in cargoes	Modification	Application	Clinical stage	Reference
**Lipid-based NPs**						
Doxil	Janssen	mRNA, Small molecule, photothermal Agent, Cyclic dinucleotide, protein, siRNA, pDNA	Surface modification, charge	Kaposi's sarcoma, ovarian cancer, multiple myeloma	Phase III	44-50
DaunoXome	Galen			Kaposi's sarcoma	Phase III	
AmBisome	Gilead Sciences			Fungal/protozoal infections	Phase III	
Onpattro	Alnylam Pharmaceuticals			Transthyretin-mediated amyloidosis	Phase III	
BNT162b2	BioNTech/Pfizer			COVID-19	Phase II	
**Polymer-based NPs**						51-59
Oncaspar	Servier Pharmaceuticals	Small molecule, Protein, gRNA, ssDNA, Anti-sense RNA, Cyclic dinucleotide, siRNA	Surface modification, Shape	Acute lymphoblastic leukaemia	Phase III	
Plegridy	Biogen			Multiple sclerosis	Phase III	
Eligard	Tolmar			Prostate cancer	Phase III	
**Inorganic NPs**						
INFeD	Allergan	Small molecule, imaging agent, Neoantigen, adjuvant, Photosensitizer, NPs for magnetic Hyperthermia, miRNA, siRNA	Surface modification	Iron-deficient anaemia	Phase III	60-65
DexFerrum	American Regent			Iron-deficient anaemia	Phase III	
Feraheme	AMAG			Iron deficiency in chronic kidney disease	Phase III	
**Carbon NPs**						
NA	Chinese University of Hong Kong, Shenzhen	RNA-Lyse, small molecular	Surface modification, charge	COVID-19, viral infectious diseases	NA	31
